# Supplementary material for: Prediction of Alzheimer’s Disease by a Novel Image-Based Representation of Gene Expression
Source: Genes (Basel). 2022 Aug 8;13(8):1406. doi: 10.3390/genes13081406 (PMC9407775; doi:10.3390/genes13081406)
Supplement: Supplementary file 1 [file genes-13-01406-s001.zip › genes-1844413-supplementary.pdf]

Table S1. Alzheimer's disease detection studies based on gene expression data. AUC (Area under the curve); Acc (Accuracy); DNN (Deep Neural Network); SVM (Support Vector Machine; RF (Random Forest); CFG (Convergent Functional Genomics); LASSO (Least Absolute Shrinkage and Selection Operator); PCA (Principal Component Analysis); t-SNE (t-distributed Stochastic Neighbor Embedding); RFE (Recursive Feature Elimination);

Study	Dataset	Number of Samples	Number of Genes	GS Method	Number of Selected Genes	ML Model	Performance
Lee et al. [21]	GSE63060	AD:145, CTL:104	7584	CFG	353	DNN	AUC: 0.874
	GSE63061	AD:139, CTL:134	6154		188	SVM	AUC: 0.804
	ADNI	AD:63, CTL:136	3897		922	DNN	AUC: 0.657
Li et al. [23]	GSE63060 + GSE63061	AD:245, CTL:182	16,928	LASSO	3601	SVM	AUC: 0.859<br>Acc: 0.781
Wang et al. [41]	GSE5281	AD:87, CTL:74	23,643	t-test	1001	SVM	AUC: 0.894
Park et al. [42]	GSE33000 + GSE44770	AD:439, CTL:257	19,488	PCA	35	RF	AUC: 0.531,<br>Acc: 0.624
				t-SNE	35	SVM	AUC: 0.511,<br>Acc: 0.632
Voyle et al. [38]	GSE63061 + DCR	AD:118, N:118	261	RFE	12	RF	AUC: 0.724,<br>Acc: 0.657

Table S2. Demographic overview of the datasets.

	GSE_63060		GSE_63061		GSE_140829	
**Classes**	**N (M/F)**	**Age years+- STDEV**	**N (M/F)**	**Age+- STDEV**	**N (M/F)**	**Age years+- STDEV**
**AD**	145 (46/99)	75.4+-6.58	139 (54/85)	77.89+-6.67	204 (100/104)	73.0+-7.09
**MCI**	80 (41/39)	74.45+- 6.00	114 (46/68)	78.39+-7.38	134 (72/62)	73.2+-6.99
**CTL**	104 (42/62)	72.375+- 6.34	135 (54/ 1)	75.41+-6.17	249 (110/139)	73.6+-6.25
